# Advancing protein biosensors: redefining detection through innovations in materials, mechanisms, and applications for precision medicine and global diagnostics

**DOI:** 10.1039/d4ra06791f

**Published:** 2025-04-11

**Authors:** Kanchan M. Joshi, Sanyukta Salve, Datta Dhanwade, Manisha Chavhan, Smita Jagtap, Manish Shinde, Ravina Holkar, Rajendra Patil, Vasant Chabukswar

**Affiliations:** a Nowrosjee Wadia College Pune 411007 MH India; b Institute of Bioinformatics and Biotechnology, Savitribai Phule Pune University Pune 411007 MH India; c Additive Manufacturing & Advanced Materials - Electronics & Energy (AM2-E2), Centre for Material for Electronic Technology (C-MET) Panchawati, Off Pashan, Road Pune 411008 MH India; d Department of Biotechnology, Savitribai Phule Pune University Pune 411007 MH India rhpatil76@gmail.com

## Abstract

Protein biosensors are significant tools in modern diagnostics due to their exceptional sensitivity and specificity in detecting protein biomarkers critical for disease diagnosis, therapeutic monitoring, and biomedical research. Innovations in transduction methods, nanomaterials, and point-of-care system integration have spurred recent advancements in biosensor technology. This summary examines key developments in protein biosensors, focusing on their structure, applications, and future potential. Nanomaterial-enhanced electrochemical biosensors, such as graphene, polyaniline, and carbon nanotubes, offer improved signal transmission due to their large surface area and faster electron transfer rates. Label-free immunosensors activated with gold nanoparticles and MXene-based sensors capable of combined biomarker analysis for detecting ovarian cancer are notable examples. During the COVID-19 pandemic, colorimetric and fluorescence optical biosensors facilitated easier diagnostics. An example of this is the incorporation of SARS-CoV-2 detection technologies into mobile phones. Real-time, label-free tracking with molecular precision is now possible due to the development of new methods, such as CRISPR-based platforms and quartz crystal microbalance (QCM)-based biosensors. This advancement is crucial for effectively managing infectious diseases and cancer. Synthetic fluorescence biosensors increase diagnostics by improving the visualization of protein interactions and cellular communication. Despite these achievements, challenges related to scalability, sustainability, and regulatory compliance remain. Proposed solutions include sustainable biosensor manufacturing, artificial intelligence-enhanced analytics for efficacy evaluation, and multidisciplinary approaches to optimize interaction with decentralised diagnostic systems. This work demonstrates how protein biosensors can advance precision medicine and global health.

## Abstract 2

Herein, an overview of biosensors, outlining their mechanism, types, and applications is provided. It illustrates analyte interaction with target proteins and ensuing signal transduction and applications in healthcare, biotechnology, and food safety.

## Graphical abstract

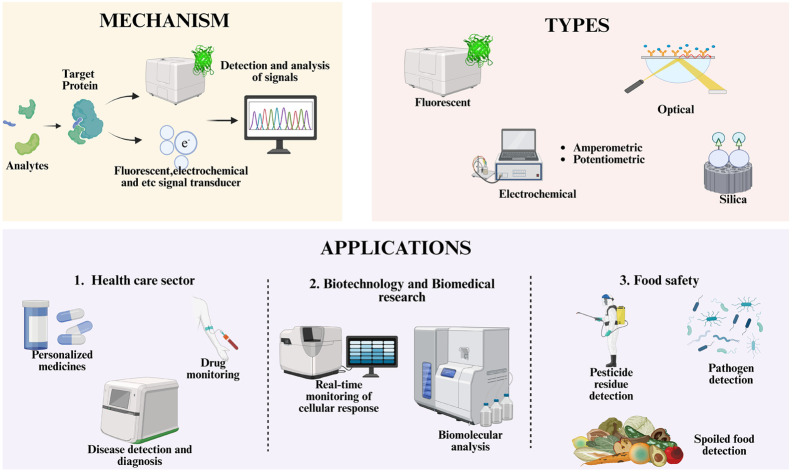

## 1 Introduction

Over the last two decades, health-related issues have considerably increased worldwide. This trend is also reflected in a recent report published by the WHO in May 2023, which highlights more than 50 health-related indicators, such as infection diseases, universal health coverage, health systems, and environmental risks. The report also states that approximately 3.6 billion people globally live in extremely susceptible conditions due to the effects of climate change, water pollution, lack of water, sanitation, and hygiene (collectively referred to as WASH), air pollution, and unintentional poisoning (World health statistics 2023). Medical treatment costs to tackle these factors are already very high (billions of dollars annually), and these costs are further increased due to improper or delayed diagnoses, which also reduce the quality of human life. The human body is composed of protein biopolymers, which are important for cell membrane formation and serve as important micronutrients. These proteins have a specific three-dimensional structure, determined by the arrangement of atoms in an amino acid chain. In a protein molecule, amino acids are linked together by peptide bonds between the carboxyl and amino groups of neighbouring residues. The sequence of amino acids in a protein is defined by the sequence of a gene, which is encoded in the genetic code. However, a change in the genetic code sequence is called a mutation, and most cancers begin when one or more genes in a cell undergo a mutation.^1^ The disruption of cell regulation leads to changes in important genes, which result from mutations in the DNA sequence of chromosomes. Mutations can be very small changes but may affect few or many nucleotides, causing major changes in the structure of chromosomes.^1^ Therefore, the determination of mutation at the initial stage is very important to seek early medical treatment, which can be achieved by the biosensor, a small electronic gadget, useful for providing faster, cheaper and simpler nucleic-acid assays. It brings together the biochemical molecular recognition properties on the basis of selective analysis, which incorporates the biological recognition element with a physical transducer. The first biosensor was reported by Clarks in 1956 for oxygen determination.^2^ Then, an amperometric enzyme electrode for glucose determination was developed in 1962, as a glucometer for electrochemical detection of oxygen or hydrogen peroxide by using a glucose oxidase electrode.^3^ The first fiber-optic biosensor was described in 1975 by Lubbers and Opitz.^3^ Thereafter, many incredible developments occurred in biosensing technology, which helped to improve the human life. Yet, there are many challenges associated with biosensor development in the bimolecular, biomedical, medical and healthcare fields, such as chronic diseases, women health issues, cancer determination. Therefore, the urgent need remains for rapid, reliable, specific, and sensitive methods for diagnosis of cancer markers and related health conditions at an early stage. In the future, a biosensor, as the device which converts a biological response to an electrical signal, will not only become even more important but also an indispensable part for biomolecule detection, and biomedical diagnostics.^4,5^

## 2 Biosensor mechanism

A biosensor system mainly involves three parts: analyte recognition (bioreceptors or biological recognition elements), signal transduction, and readout. The bioreceptor can be an organism, tissue, cells, enzymes, antibodies, and nucleic acids, while the transducer can be electrochemical, optical, thermal or mechanical. Nucleic acid-based biosensors possesses sequence variability and specificity, drawing significant attention from researchers for their development. Nucleic acids are composed of single-stranded (ssDNA), which can hybridize with a complementary strand for exceptionally high efficiency and specificity. This characteristic allows the detection of complementary DNA or RNA strands to be easily performed in such sensors. DNA aptamer technology enables the recognition of complementary strands, metal ions, small molecules, proteins, and even cells. Conversion of DNA binding events into useful biosensors often requires sensor immobilization. It is very difficult to screen for genetic disorders caused by base-pair mutations, and early-stage diagnosis using nucleic acid-biosensors is crucial.^6,7^

Nucleic acid-based biosensors exploit the inherent specificity of nucleic acid hybridization, where ssDNA or RNA pairs precisely with its complementary sequence. This property allows the detection of specific DNA or RNA targets, making these biosensors particularly effective in genetic disorder screening and early-stage diagnostics. For example, DNA aptamers—synthetic oligonucleotides—can bind not only to complementary strands but also to diverse targets, including metal ions, small molecules, proteins, and even entire cells. Aptamers offer high selectivity and can convert binding events into measurable signals *via* immobilization onto sensor surfaces, enhancing their stability and reusability.

A critical application of nucleic acid biosensors is in detecting microRNAs (miRNAs), short noncoding RNAs (19–25 nucleotides) involved in gene regulation, tumor initiation, metastasis, and apoptosis.^8^ Aberrant miRNA expression profiles are hallmarks of various human cancers, making them valuable clinical biomarkers.^9–11^ Conventional miRNA detection methods, such as Northern blotting, have been complemented by modern biosensors capable of dynamic and modular responses. These devices, often designed as molecular switches, transition between a closed (dark) state and an open (luminescent) state upon analyte binding. This thermodynamically coupled mechanism enhances sensitivity, allowing precise detection of genetic mutations, infectious agents, or cancer biomarkers.^12^

Nucleic acid biosensors are also pivotal in fields beyond clinical diagnostics. For example, their specificity in detecting viral RNA has proven invaluable in pandemic response systems, enabling rapid and accurate pathogen identification. Similarly, aptamer-based platforms are being used for therapeutic drug monitoring, detecting small-molecule drugs in blood or tissue samples with unparalleled precision.

Membrane protein-based biosensors capitalize on the functional and structural roles of proteins embedded within biological membranes. These biosensors are classified into two distinct platforms: lipid bilayer-based systems and cell-based systems.

• Lipid bilayer-based platforms: these systems incorporate membrane proteins into synthetic lipid bilayers, which act as intermediaries between the protein and the sensor device. Such setups maintain the native environment of membrane proteins, preserving their structural integrity and functionality. The lipid bilayer serves as a critical interface, enabling the detection of analyte binding events through signal transduction mechanisms such as changes in impedance, fluorescence, or mass. These biosensors are extensively used for detecting specific proteins, such as HER2 receptors, which are critical in cancer diagnostics.^13^

• Cell-based platforms: in this approach, membrane proteins are expressed in living cells, organoid models, or whole organisms. These platforms measure dynamic biological responses, such as ligand–receptor interactions or the effects of pharmacological agents, by capturing functional information rather than static molecular measurements. While these systems may lack the environmental sensitivity of molecule-based biosensors, they provide invaluable insights into sample toxicity and drug pharmacology.^14,15^

Applications of membrane protein-based biosensors are diverse. For example, they are instrumental in detecting critical proteins such as anti-apoptotic BCL-2, botulinum neurotoxin B, and cardiac troponin I. These biosensors are widely used in pharmacological studies to evaluate drug–ligand interactions, offering real-time assessments of drug efficacy and toxicity. In cancer research, membrane protein-based systems have been employed to detect circulating tumor markers and monitor cell signaling pathways. Furthermore, the ability of these biosensors to mimic physiological environments makes them indispensable in testing novel therapeutics and studying receptor-mediated cellular responses.^13–15^

### 2.1 Types of biosensors

#### 2.1.1 Electrochemical biosensors

**Fig. 1:**
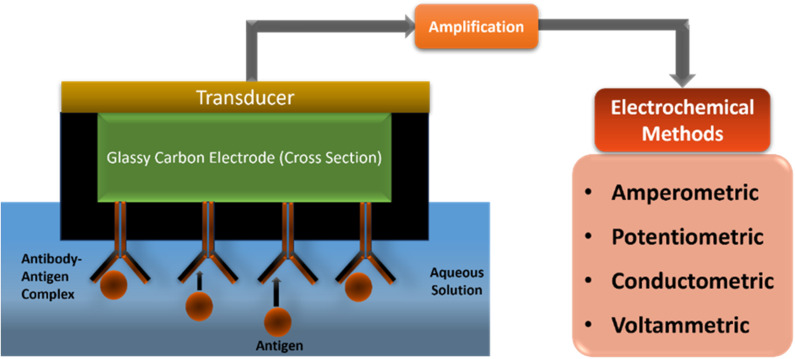
Schematic of development of an electrochemical biosensor.

To prepare electrochemical biosensors, the surface of metal and carbon electrodes are modified by using various biomaterials such as enzymes, proteins, nucleic acids, tissues, receptors, antibodies or DNA (Fig. 1). An output signal is generated upon specific binding or catalytic reactions of biomaterials on the electrode's surface.^16^ In the biosensor mechanism, electrodes selectively react with the target molecule, where the biocatalytic devices convert chemical energy into electrical energy through redox reactions. During the sensing process, a known voltage is applied to the electrode to measure the redox potential, where the change in voltage is measured in the form of response and recovery time. The read-out device measures the change in current values.^17^

Hence, in an electrochemical biosensor, direct monitoring of the formation of an antibody–antigen conjugated layer takes place with many potentials. The advantages are a higher signal-to-noise ratio, ease of detection, lower assay cost, faster assays, and shorter detector response times.^17^

Biosensors are used for different transduction techniques and for fast screening strategies. The advanced biocompatible materials and electrocatalytic nanomaterials have extended and enhanced the field of biosensors. For example, in an iron complex molecule, the redox properties of ferroceneboronic acid (FcBA) and the ferrocene-sugar adduct are different because FCBA and its derivatives have electron-withdrawing properties, so that it binds to 1,2 or 1,3 diol residues of sugar to form cyclic boronate ester bonds.^18^ Therefore, FcBA and its derivatives are used for electrochemical sensors in biomedical fields, including diabetes diagnosis and hormone analysis.^2,19^ Hence, it is important to place special emphasis on the selection of receptors in the biosensor development.^20^

Depending on the working principle of a biosensor, electrochemical biosensors are classified into different types, such as amperometric, potentiometric, conductometric and voltammetric. In conductometric detection, the specific conductance of a material is measured and can be applied for both electroactive and electroinactive materials. In a chemical reaction, conductivity of the sample solution changes according to the change in solution composition and medium.^21^ However, in the presence of enzymes, it provides a charged product by changing the ionic strength of the reaction mixture, which results in increased conductivity. The potentiometric sensor works on the principle of the Nernst equation. It measures the concentration of analytes by changing the potential of electrochemical cell during the redox reaction. It includes two reference electrodes which are sensitive to ions that belong to the sensor, to measure the potential across a membrane.^17^ In a potentiometric biosensor, a biological sensing element is attached to a physicochemical transducer to provide electrical potential as an analytical signal.^21^ The plants contain some polyphenolic and polyhydroxy compounds, such as lignin based flexible piezoresistive materials, carbonized lignin, and polydimethylsiloxane (CL/PDMS) that show high sensitivity for the measurement of the pulse rate and strength where high sensitivity is observed as 57 kPa and a stable response is observed at 0.1 to 2.5 Hz frequency (Table 1).^29,30^

**Table 1:** Comparative analysis of biosensor performance: materials, sensitivity, response time, and cost-effectiveness

Sr. no.	Material and method	Sensitivity	Response time and cost effectiveness	Ref.
1	Semiconductor-based sensing platform, light-addressable potentiometric sensor (LAPS) for detection of carcinoembryonic antigen (CEA) from a CEA-producing human colon cancer cell line. It detects protein cancer biomarkers	Simple, highly sensitive and specific	Response range: 2.5–250 ng mL^−1^; inexpensive	22
3	Chit/Ti_3_C_2_T_*x*_	Sensitivity for cholesterol with a low detection limit and high sensitivity of 132.66 μA nM^−1^ cm^−2^	Limit from 0.3 to 4.5 with low detection limit of 0.11 nM, excellent practicability, favourable selectivity and stability	23
4	Thin-film multiplexed electrodes with b2LOxS and DET-type glucose dehydrogenase	Lactate sensitivity: 4.1 nA mM^−1^ mm^−2^. Glucose sensitivities: 56 nA mM^−1^ mm^−2^	Linear range for lactate: 0.5–20 mM. Linear range for glucose: 0.1–5 mM; inexpensive	24
5	Single-walled carbon nanotubes screen-printed electrodes (SWCNT-SPEs), sensitive for Peptide Nucleic Acid (PNA)	Higher sensitivity at LOD of 71 pM and a LOQ of 256 pM for GC-SPEs were 430 Pm and 1.43 nM	Good response	25
6	Single-walled carbon nanotubes (SWCNTs) and (PANI) as transducing elements for protein detection. These are two different solid-contact selective potentiometric thrombin aptasensors	Both sensors have similar limits for detection of proteins	Detection range: 2.97 mV per decade and 8.03 mV per decade for the PANI and SWCNTs aptasensors. Lower noise level	26
7	The surface of single-walled carbon nanotubes (SWCNTs). The biosensor was prepared by binding Aβ-42 to the SWCNT surface, then imprinting it by adding acrylamide (monomer), *N*,*N*′-methylene-bis-acrylamide (crosslinker), and ammonium persulphate (initiator)	Detection of amyloid β-42 (Aβ-42) in point-of-care analysis	Cationic slopes: 75 mV per decade in buffer pH 8.0. Detection limit: 0.72 μg mL^−1^. Simple design, low response time, good selectivity and cost-effective	27
8	Using self-assembled monolayers of alkanethiol with hydroxyl terminal groups as the matrix material, the sensing layer was created on the surface of the gold-coated silicon chip-an electrochemical transducer for hemoglobin detection	It is highly sensitive for hemoglobin, myoglobin and ovalbumin	Potentiometric measurement in DPBS (Dulbecco's phosphate buffered saline) at pH 7.15 ± 0.1. Isoelectric point of myoglobin, hemoglobin and ovalbumin is 7.0, 6.8 and 4.6, respectively. Higher re-occupation percentages of reception sites, fast response, integration of sensing element and transducer	28

#### 2.1.2 Optical/visual protein sensor

**Fig. 2:**
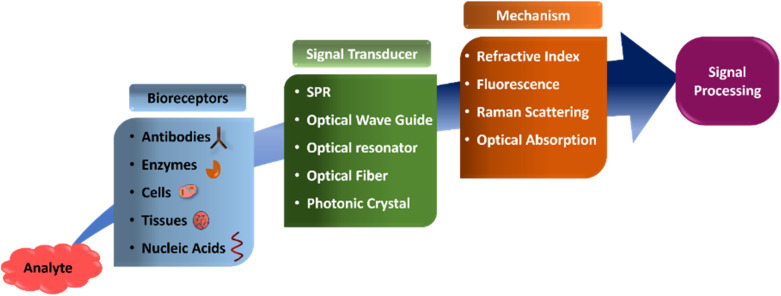
Optical biosensor flow-sheetdiagram.

The main objective of the biosensor is to produce a signal which is directly proportionate to the concentration of the analyte. It is based on a colour change of the reaction mixture, and concentration can be determined by measuring absorbance, reflectance, luminescence and fluorescence. In an optical biosensor, selection of a recognizable element is crucial because in an affinity reaction, molecular recognition depends on affinity and specificity to give a stable complex. The recognition from a biological entity must be converted to a distinguishable electrical signal, optical signal or thermal signal by the means of a transducing unit is important. Therefore, optical biosensors are capable of detecting a bimolecular complex, converting the physical or chemical signal to an optical signal or electrical signal which can be further processed for the determination of the concentration of the analyte^31^ (Fig. 2). Recently, researchers have proposed highly selective microbial biosensors which were constructed by inducing a desired microbe metabolic pathway depending on the condition of cell culture. However, genetically engineered microorganisms provide selectivity and sensitivity to the microbial biosensor at the DNA level.^32^

However, in the sensor development process, sensitivity (*S*) and detection limit (DL) are two important parameters to enhance the performance of the biosensor. Sensitivity is defined as the strength of incident light that the matter interacts with, to the magnitude change of the transduction signal in response to the change of analyte concentration. In most evanescent-based biosensors, sensitivity is dependent on the fraction of the optical wave in the bulk solution or the light intensity on the sensor surface. The DL is defined as the smallest detectable amount of analyte and is determined by the resolution of a read-out system. Nevertheless, DL is strongly related to the noise arising from the light source intensity fluctuation, temperature fluctuation, *etc.* Taking into account overall noise, the DL can be expressed as the minimum resolvable signal DL = *σ*/*S* where *σ* is the noise in the transduction signal. A higher sensitivity is associated with the noise, which produces a moderate DL. There are three ways to specify the DL of a biosensor. (i) For bulk sensing, DL as a refractive index unit (RIU) is used to quantify the biosensor performance. (ii) For surface sensing, surface analyst mass density or total mass in a unit of RU (resonance units 1 pg mm^−2^) reflects the intrinsic sensing capability of a transducer. (iii) Here the analyte concentration is used in a unit such as ng mL^−1^ or molarity, and it is quite easy to determine as no information on the surface mass density is required. However, DL values of different formats are correlated and the best value for bulk index is found in the range of 10–6 to 10–8 RIU. Hence, such biosensors can determine the lowest concentrations (ng ml^−1^ or pg ml^−1^) of analyte samples.^33^ The optical-based biosensor with a combination of fluorescence and small molecule/nanomaterial has been widely accepted. In the mammalian cells, quantification of kinase activity is of particular interest for signal transduction studies because more than one third of cellular proteins have been identified to possess phosphorylation sites. Among these kinases, mitogen activated proteins kinases (MAPKs) play a central role in the signalling network of cells (Table 2).^42^

**Table 2:** Comparative analysis of biosensor performance: materials, sensitivity, response time, and cost-effectiveness

Sr. no.	Material and method	Sensitivity	Response time and cost-effectiveness	Ref.
1	Q-tip fabricated colorimetric-biosensor with 1-ethyl-3-(3-dimethylaminopropyl) carbodiimide hydrochloride (EDC) and *N*-hydroxysuccinimide (NHS) for SARS-CoV-2 detection	Sensitive (100 pfu mL^−1^); working range: 10^3^–10^8^ pfu mL^−1^ for SARS-CoV-2. Linear range of quantitation: 103–108 pfu mL^−1^	Fast response within 5 min, rapid, and relatively cheap for onsite use	34
2	Transcriptional biosensor system for 293/hTLR4A-MD2-CD14pGL4.26-mcherry-NF-κB cells (bacterial lipopolysaccharide (LPS))	0.01 ng mL^−1^	Highest response. Low cost and easy to perform	35
3	Heart-on-a-chip measuring excitation and contraction in a single tissue	Voltage sensitivity dye RH237 at 4 mM	Response time: 2 min	36
4	*In vitro* assessment of cytotoxicity based on electrochemical impedance spectroscopy (EIS), measuring frequency-dependent impedance data of the cell monolayer and its analysis with a theoretical cell–electrode model	0.1 μM	Activation time: 2 min	37
5	Evanescent wave optics fibre immunosensor using kinetic and optical methodology	40 pM or 5 ng mL^−1^ antigen concentration	Response time: >1 min, cost- effective	38
6	Confocal optics-based sensor for detection of interleukin-8 (IL-8), an oral cancer marker, in buffer solution	LOD: 1.1 pM	60 min	39
7	Streptavidin-coated glass cover slips of optical protein micro-sensor for interleukin-8 (IL-8) protein, an oral cancer marker	Detection limit: 1.1 pM (particulate matter)	40 min	40
8	QCM biosensor for human serum albumin detection	0.026 μg mL^−1^; linear range: 0.05 and 0.500 μg mL^−1^	High response	41

#### 2.1.3 Silica, quartz/crystal and glass biosensors

Silica, quartz and glass materials are transparent materials having unique electrochemical properties. Additionally, silicon nanomaterials have greater potential for technological advances in biosensor applications because it presents non-toxicity, biocompatibility and abundance, with excellent electronic, optical and mechanical properties. Hence, it provides an important precondition for biomedical and biological applications such as bioimaging, biosensing and cancer therapy. In molecular adsorption, quartz crystal microbalance with dissipation (QCM-D) is a real–time nanogram-accurate technique for analysing various processes on biomaterial surfaces. It provides information of amount on the mass deposited and rate of deposition on a film by real time change in frequency, because the change in mass of such films is linearly related to the change in the oscillation frequency. Quartz crystal microbalance (QCM) biosensors are based a molecular imprinting technique where a synthetic recognizable polymeric material is used. Hence, this technique results in quick detection of biomolecules.^41^ However, silicon nanowires in combination with gold nanoparticles are used as effective materials in cancer therapies.^43–45^ Silica nanomaterial with fluorescence is used for the determination of circulating tumor cells up to 1–10 cancer cells out of 100 cancer cells.^46^ Quantitative measurement of protein by a non-contacting *i.e.* wireless-electrodeless multichannel (WE-MQCM) method is used where vibrations of quartz oscillators are detected by antennas *via* electromagnetic waves. This is a cost-effective and biosafe technology for improving bioinstrumentation for biomedicine technology (Tables 3 and 4).

**Table 3:** Comparative analysis of biosensor performance: materials, sensitivity, response-time, and cost-effectiveness

Sr. no.	Material and method	Sensitivity	Response time and cost effectiveness	Ref.
1	Piezoelectric biosensor of gold-coated QCM crystals (QSX 301) with a 100 nm gold layer and a 5 nm chromium adhesion layer. Single-step label-free hepatitis B virus detection	Sensitivity up to fmol cm (10^−15^ moles)	Fast response	47
2	Quartz crystal detection chips with gold and reduced graphene oxide (rGO)-coated surfaces functionalized with bovine serum albumin (BSA) *via* biomolecular interaction with a typical bovine protein	Custom quartz crystal microbalance (cQCM): bare Au ∼1.63 (Hz mg^−1^ mL^−1^), rGO ∼0.10 (Hz mg^−1^ mL^−1^), QSense: bare Au ∼0.14 (Hz mg^−1^ mL^−1^), rGO ∼0.01 (Hz mg^−1^ mL^−1^)	2 min	48
3	Piezoelectric biosensor using lead titanate zirconate (PZT) ceramic resonator as the transducer for detecting cancer biomarkers, such as prostate-specific antigen (PSA) and α-fetoprotein (AFP)	High sensitivity: 0.25 nm mL^−1^	30 min; cost-effective	49
4	Polymers (MIPs) employed as recognition elements in biomimetic sensors for PoC testing of cardiovascular disease-related biomarkers	10.2 ng L^−1^	10 min	50
5	QCM for classical swine fever (CSFV) detection	4–21 μg mL^−1^ CSFV. LOD: 1.7 μg mL^−1^, with a resonance frequency response of about 38 Hz μg^−1^ mL^−1^	—	51
6	QCM MIP QCM electrode with linearity of 99.53% in the concentration range of 50–1000 ng mL^−1^	Limit of detection (LOD): 2.3 ng mL^−1^, limit of quantification (LOQ): 7.8 ng mL^−1^ for melamine	Fast response	52
7	QCM device for detecting specific 10-mer DNA chains		(10 Hz/10 min)	53
8	Aptamer-based QCM for cell detection	Sensitivity: 2 × 10^3^–1 × 10^5^ cells per mL	Response limit: 1160 cells per mL. It is a simple, rapid and economical method	54

**Table 4:** Comparative analysis of biosensor performance: materials, sensitivity, response time, and cost-effectiveness

Sr. no.	Material and method	Sensitivity	Response time and cost effectiveness	Ref.
1	2D graphene. The portable 3D paper-based analytical device possessed a wide calibration range of 0.001–10 ng mL^−1^	Low detection limit of 0.5 pg mL^−1^; limit: 9 nM	Fast response (2.92 s)	55
2	Electroanalytical performance and sensitivity of screen-printed electrodes (SPE) with Au nanoparticle-based biosensing	0.25 fg mL^−1^	Device demonstrated LOD of 90 fM and 10–30 s response time	56
3	Graphene oxide integrated conducting paper with poly (3,4-ethylenedioxythiophene): poly(styrenesulfonate) (PEDOT:PSS) and carbon nanotubes	Sensitivity: 7.8 μA (ng ml^−2^ cm^−2^)	Linear detection range: 2–15 ng mL^−1^	57
4	Microfluidic paper-based analytical device (μPAD) using the horseradish peroxidase (HRP)-*O*-phenylenediamine-H_2_O_2_ electrochemical detection system	Greatly enhanced sensitivity	Exhibited good response, stability, reproducibility, and accuracy	58
5	Nanomaterial-based biosensor with five signal transducing methods for bacteria cells, toxins, mycotoxins, and protozoa cells	Limited sensitivity	Total response time may reach 10 min to 2 h	59
6	Ag_2_SnO_3_/GCE biosensor for sorafenib detection in human serum samples	Linear range: 0.49 to 167.41 μM; sensitivity: 0.988 μA μM^−1^ cm	Good response	60
7	2D transition metal dichalcogenide (TMD)-based FETs, including CNTs, graphene, rGO, 2D transition-metal carbides (MXene), and graphene/MXene heterostructures	0.01XPBS	Response time: 1 s at 1 ng mL^−1^	61

#### 2.1.4 Nanomaterials-based biosensors

Nanomaterials are used as transducers in biosensors, which has led to the emergence of novel and multifunctional nanotechnologies. In PoC testing, nanomaterials are being increasingly used.^62^ A microfluidic platform integrated with graphene-gold nanocomposites as an aptasensor for detecting norovirus with *z* detection limit 100 pM was reported by Chand *et. al.*^63^ Similarly, a broad range of nanomaterials are used as transducers in biosensors to enhance sensing activity, such as gold, silver, copper, carbon nanotubes, carbon nanofibers, fullerenes and their composites^43,62,64–67^ Nanomaterials undergo redox reactions rapidly, leading to greater sensitivity and specificity than other materials.^68,69^ Platinum-based nanoparticles show a single label response for the detection of low concentrations of DNA.^70^ Quantum dots technology is used, not only in understanding the tumor microenvironment for therapeutics, but also in the delivery of nanomedicine. Semiconductor quantum dots have interesting optical and magnetic properties and hence can be efficiently linked with tumor and targeting ligands such as monoclonal antibodies, peptides, or small molecules, to target tumor antigens.^71–73^

#### 2.1.5 Synthetic fluorescent biosensors

Synthetic fluorescent biosensors measure chemical and biological process occurring in the cell in normal and pathological conditions. In methodological encoded fluorescent biosensors, proteins are used to allow for the real time monitoring of molecular dynamics in spaces. It gives proper functioning and regulation of complex cellular processes. Different types of molecular events are required different sensing strategies which are applied for the significant determination of FP-based biosensors. For example, translocation, fluorescence resonance energy transfer (FRET), reconstitutions of split FP, pH sensitivity, maturation speed, *etc.*^74^ The development of tagged biosensor using genetically encoded or synthetics fluorescence cover the way to understand the biological process including various molecular pathway inside the cell.^75–77^ However, the fluorescent biosensor is constructed according to the recognition elements, particularly for specific binding proteins, nucleic acids and aptamers while the output signal determines the concentration of target molecules.^78^ For live-animal imaging, sensor technology is used that include small angle X-ray scattering for calcium sensors and fluorescence. Resonance energy transfer probes for kinase sensing are being cited as the best biosensor method in modern physiology.^79^ Complete information of genome (genome is all genetic information of an organism) consists in nucleotide sequences of DNA. The genome includes both the genes and the non-coding DNA, as well as mitochondrial DNA and chloroplast DNA required fabrication of new biosensors. The bioluminescent sensors are attractive for light-sensitive sensing applications including optogenetics and long-term continuous monitoring because it is used to control the intermolecular complementation of split luciferase. For example, Ca^2+^ induced interaction between calmodulin and M13 drives the intermolecular complementation of split NanoLuc luciferase.^78^ NanoSwith is the protein biosensor made by the modularized luciferase. NanoLuc allows direct detection of antibodies in 1 μl serum in 45 min without washing steps with S/N ratio of 33-fold and 42-fold. Moreover, it detects SARS-CoV-2, protein of hepatitis virus (HCV) and 42-fold, gp41 of the human immunodeficiency virus (HIV) by assay clinical sample (Table 5).^86^

**Table 5:** Comparative analysis of biosensor performance: materials, sensitivity, response time, and cost-effectiveness

Sr. no	Material and method	Sensitivity	Response time and cost-effectiveness	Ref.
1	Cytosolic NADH-NAD+ redox state by combining a circularly permuted GFP T-Sapphire	pH-sensitive	Cytosolic NADH-NAD+ redox state by combining a circularly permuted GFP T-Sapphire	80
2	RNA riboswitch-based biosensor with dual fluorescence	High sensitivity up to 60 000 (a.u.)	Response detected at 40 000 (a.u) For low concentrations of naringenin	81
3	Synthetic fluorescent substrate for human MAPKs (ERK, JNK and p38). To monitor MAPK activity in mammalian cells	Sensitivity is highly dependent on the specificity of the antibody and sample preparation	50 ng mL^−1^	42
4	Fluorescent biosensor based on cyclic signal amplification technology for detection of nucleic acids, proteins, enzymes, adenosine triphosphate (ATP), metal ions, and exosomes	High sensitive and specificity	Can induce local inflammatory response and is low cost	82
5	Fluorescence of VS QDs. A VS_2_ QD/MoS_2_ nanosheet-based fluorometric TET aptasensor	High sensitivity, low detection limit, and high specificity. Biosignal analysis in the linear range of 1 to 250 ng mL^−1^	0.06 ng mL^−1^	83
6	Ruthenium(ii) tris(bipyridyl) co-ordinate complex	Sensitive at 10 μM. Senses and discriminates proteins, including therapeutically relevant targets, hDM2 and MCL-1, using linear discriminant analysis (LDA)	Response detected immediately after incubation at 2 h and 20 h	84
7	Caspase activity sensor by polarization anisotropy multiplexing (CASPAM)	Sensitive for enzymatic activity	Heterogeneous and complex responses made visible by simultaneous imaging of different biosensors at 10 s	85

### 2.2 Emerging biosensors for protein detection

Protein detection biosensor development has advanced significantly as a result of novel materials and transducer routes that improve sensitivity, selectivity, and usability. Recently, a wide variety of biosensor types have surfaced, each using distinct materials and techniques to improve protein identification in samples that are challenging to identify. The most advanced novel biosensor technologies are discussed in this section, along with case studies that highlight their benefits and real-world uses. By using conductive materials like graphene, polyaniline, and carbon nanotubes to augment electrochemical signals, nanomaterial-enhanced electrochemical biosensors have shown significant increases in detection sensitivity. These sensors have been modified to maximise surface area and electron transfer rates in order to improve the signal response to certain specific protein biomarkers.

Researchers focused on creating a label-free electrochemical immunosensor for ovarian cancer detection that recognises the cancer antigen CA125 using gold nanoparticles (AuNPs) and multi-wall carbon nanotubes (MWCNTs). By adding carboxylic groups to the MWCNTs, the acid treatment increased their surface area and conductivity, improving AuNP immobilisation and the sensitivity of detection down to 0.001 μg mL^−1^. The MWCNT-AuNP-based sensor highlights how structural alterations in carbon nanomaterials can significantly enhance early cancer detection performance, and provides a potential, affordable method for point-of-care ovarian cancer diagnostics.^87^

A study was carried out on similar research on the application of the 2D nanomaterial Ti_3_C_2_ MXene in the creation of electrochemical biosensors intended especially for the identification of cancer biomarkers. MXenes like Ti_3_C_2_T_*x*_ enhance biosensor performance by better immobilising bioreceptors such as DNA aptamers and antibodies. The study shows that MXene-based sensors have low detection limits and high sensitivity. These sensors' capacity to multiplex and detect numerous cancer indicators at once adds to their therapeutic value and makes them especially well-suited for early-stage cancer diagnosis.^88^

### 2.3 Optical biosensors for viral and cancer biomarker detection

Optical biosensors have evolved tremendously, particularly those using colorimetry and fluorescence. When target proteins are identified, these biosensors produce optical signals such as colorimetric changes or fluorescence, allowing for fast, non-invasive diagnosis. They are essential for point-of-care applications, particularly in the detection of infections and cancer biomarkers, because of their effectiveness, interpretability, and compatibility with mobile devices.

The COVID-19 pandemic has highlighted the importance of portable, cost-effective diagnostic technologies with high sensitivity. Optical biosensors have proven to be quite effective, especially those including plasmonic nanoparticles such as gold nanoparticles (AuNPs). These biosensors improve accessibility in areas with limited resources by allowing for fast detection and seamless integration with smartphone technology.

In order to demonstrate this, researchers developed a colorimetric biosensor that can identify SARS-CoV-2 in saliva samples. When plasmonic AuNPs functionalised with polyclonal antibodies came into contact with the virus, the biosensor displayed a significant colour shift. The integration of smartphone photography and machine-learning analysis enabled an accurate diagnosis, achieving a sensitivity of 0.28 PFU mL^−1^ and a diagnostic accuracy of 100%. The biosensor demonstrated exceptional sensitivity, distinguishing SARS-CoV-2 from other viruses, including influenza, even in intricate environmental samples like river water. Its rapid detection, minimal sample requirement, and portability make it an effective tool for pandemic management and environmental pathogen monitoring.^89^

The effectiveness of these optical biosensors highlights their transformative influence on decentralised diagnostics. Addressing cancer biomarkers requires solutions tailored to individual issues, particularly in achieving the sensitivity and specificity necessary for early diagnosis. Prostate-specific antigen (PSA) levels are often used to diagnose prostate cancer. However, this method can be flawed and give false results, which could lead to unnecessary biopsies. Attention has been drawn to biosensors that aim for more specific biomarkers, like PCA3, as a way to get around these problems. RNA-based aptamers are a good choice for electrochemical biosensors because they are very sensitive and only pick out target molecules. Researchers created a new type of electrochemical detector that targets the PCA3 biomarker. It used an RNA-based aptamer that was attached to a gold electrode surface and paired with ferrocene. The aptamer's better binding affinity made it possible to accurately detect PCA3 at concentrations ranging from 0.1 ng mL^−1^ to 1 μg mL^−1^. The inclusion of the ferrocene label significantly enhanced the electrochemical signals, resulting in distinct anodic and cathodic currents associated with PCA3 levels. This technology offers a dependable tool for the early diagnosis of prostate cancer, fulfilling a critical requirement for rapid and precise point-of-care testing. The advancements achieved with this detector demonstrate the significance of aptamer-based devices in cancer detection. The advancements in optical biosensors signify the onset of a new era of precise diagnostic testing targeting specific medical issues.^90^

### 2.4 Quartz crystal microbalance (QCM) biosensors for biomolecular analysis

Quartz Crystal Microbalance (QCM) biosensors provide precise real-time observation of biomolecular interactions, eliminating the need for labelling. Their technology uses frequency alterations to detect minor mass fluctuations on the surface of a quartz crystal. Their great sensitivity and low sample consumption make them highly suitable for applications requiring accuracy and selectivity. This is highly pertinent to cancer biomarker identification. Nanomaterials, including reduced graphene oxide (rGO) and gold nanoparticles (AuNPs) have significantly improved the performance of QCM biosensors, consequently broadening their application in oncology and therapeutic research.

In a study, noteworthy advancement was achieved through the use of naturally reduced rGO and AuNP nanocomposites to create an electrochemical biosensor designed for the detection of the liver cancer biomarker miRNA-122. The creation of rGO/Au nanocomposites *via* an environmentally benign soapnut solution has resulted in a sensor distinguished by an impressively low detection limit of 1.73 pM, as well as an extensive detection range extending from 10 μM to 10 pM.^91^

This biosensor's dependable and regular measurements make it particularly suitable for early-stage liver cancer diagnostics, where increased sensitivity is crucial for effective intervention. This advancement demonstrates the potential of green chemistry in biosensor development and highlights the versatility of rGO/Au composites in improving diagnostic precision.

Researchers have improved the capabilities of QCM biosensors to address the diagnostic difficulties related to prostate cancer, particularly in individuals with inconclusive PSA results.

One creation involved an evaporation-induced rGO-coated CTC-chip to improve the diagnostic sensitivity of PSA-based testing in the “grey zone”. This technology employs the Marangoni effect during evaporation to generate micro- and nano-wrinkled reduced graphene oxide surfaces, thereby enhancing the capture of circulating cancer cells (CTCs). The integration of PSA haematological testing with CTC detection markedly improved diagnostic sensitivity from 58.3% to 91.7%, providing a non-invasive, high-sensitivity option that diminishes the necessity for biopsies. This method highlights the significance of rGO coatings in diminishing diagnostic uncertainties and enhancing patient outcomes in prostate cancer screening.^92^

The case studies demonstrate the significant impact of QCM biosensors in oncology. Researchers demonstrate the capacity of rGO/Au composites to detect liver cancer biomarkers,^91^ whereas other highlight the efficacy of rGO coatings in enhancing prostate cancer diagnostics. The enhancements collectively illustrate the adaptability of nanomaterial-enhanced QCM biosensors in addressing significant clinical challenges with precision and innovation.^92^

### 2.5 CRISPR-based nanoparticle biosensors for on-site testing

Combining CRISPR technology with nanoparticle-based biosensors creates a novel molecular platform for the detection of proteins and nucleic acids. This method combines the remarkable precision of CRISPR-Cas9 systems with the versatility of nanomaterials like gold and silver nanoparticles to achieve high selectivity. These biosensors are designed for fast, on-site testing through the use of optical detection and isothermal amplification. They are particularly well-suited for rapid deployment in resource-limited settings. CRISPR-based biosensors are revolutionising point-of-care diagnostics in infectious disease management and public health by removing the necessity for advanced laboratory equipment.

An exemplary case is the RAVI-CRISPR system.^93^ This test utilises CRISPR/Cas12a technology in conjunction with a ROX-labeled single-stranded DNA-fluorophore-quencher (ssDNA-FQ) reporter to identify nucleic acid targets, including SARS-CoV-2 and African swine fever virus (ASFV). The device exhibits a significant colour change upon detection, facilitating straightforward visual interpretation of the results. The RAVI-CRISPR assay, designed for ease of use and mobility, requires only one tube for isothermal amplification and utilises a portable hand warmer for incubation, thus eliminating the need for complex equipment. The assay employs the MagicEye app, which uses convolutional neural networks for automated result interpretation *via* smartphone photography, enhancing precision and reliability. The combination of CRISPR technology, nanoparticle sensitivity, and smartphone connectivity establishes RAVI-CRISPR as a sophisticated platform for field diagnostics, especially in resource-constrained environments.

RAVI-CRISPR illustrates the increasing significance of CRISPR-based biosensors in tackling global health issues by combining advanced molecular biology methods with readily available detection technologies. Its applications in infectious disease diagnostics demonstrate significant potential for broad implementation within decentralised healthcare systems.

### 2.6 Synthetic fluorescent biosensors for intracellular protein monitoring

Because synthetic fluorescence biosensors enable real-time observation of molecular events with extraordinary spatial and temporal precision, they have revolutionised the study of intracellular protein interactions. These biosensors use fluorescent tags that react dynamically to specific proteins to enable accurate imaging of the signalling pathways that are active within cells. These sensors, which expose protein interactions at the subatomic level, provide critical insights into disease mechanisms such as cancer formation and immune system reactions.

This development is demonstrated by the use of computational protein design, which results in the development of the Ras-LOCKR-S sensor.^93^ The capabilities of this biosensor are increased by employing fluorescent proteins in conjunction with LOCKR (Latching Orthogonal Cage-Key pRotein) technology, which enables real-time subcellular imaging of endogenous Ras activity within the cytoplasm. Through the meticulous identification of Ras-induced signalling outputs in cancer cells, the Ras-LOCKR-S system elucidates the functional dynamics of Ras signalling within oncogenic contexts. The researchers enhanced this method by employing Ras-LOCKR-PL, a proximity labelling variation designed to identify Ras-interacting proteins within EML4-ALK oncogenic granules. This technique revealed that the SAM68 protein enhances Ras signalling activity within these granules, offering significant insights into potential therapeutic targets. The findings show how computationally generated fluorescence biosensors might improve cancer research by allowing for detailed molecular mapping of signalling networks.

Fluorescent biosensors have largely concentrated on cancer signalling, whereas aptamer-based biosensors are advancing notably in the observation of immune system activity, especially in the tracking of cytokine responses.

Researchers tackled this requirement by creating a biosensor using structure-switching aptamer-modified magnetic nanobeads for the ongoing and real-time observation of interferon-gamma (IFN-γ). This platform uses a ferrocene-labeled aptamer that specifically binds to IFN-γ, initiating a structural change that deactivates the electrochemical signal. The biosensor, integrated into a microfluidic system, demonstrated an ultra-sensitive detection range of 10–500 pg mL^−1^, with a detection limit of 6 pg mL^−1^, facilitating accurate monitoring of IFN-γ in complex biological media. The remarkable tracking power of the design for dynamic immune responses supports clinical research of immunological function and cytokine activity.^94,95^

These advances show how biosensors might change biological research. Whereas researchers show how synthetic fluorescence biosensors enable complex mapping of cancer signalling networks,^93^ the aptamer-based platform emphasises the part biosensors play in real-time immunological surveillance.^94^ These developments, collectively offer vital tools for improving therapeutic and diagnostic research.

### 2.7 Technological comparison of biosensors

In this section, we compare the various types of parameters in different biosensors such as technology, specificity, detection time, linear range, analysis time, cost, portability, *etc.* Electrochemical sensors with high throughput methods focus on detection limit, analysis time and portability providing large-scale consumer markets for inexpensive biosensors for glucose and pregnancy tests using antihuman chorionic gonadotropin immobilization strips with lateral-flow technology. In this technique, the working electrode of the biosensor is based on a graphite rod (GR) electrode, which is modified with 1,10-phenanthroline-5,6-dione (PD) and glucose oxidase (GOx). The PD and GOx are layer-by-layer adsorbed on the GR electrode surface. Such biosensors show high anti interference ability to uric and ascorbic acids. Here, the lateral flow technology allows direct delivery of sample to desired spot in order to create specific interactions instead of random.^96^ The advantages of electrochemical sensors are sensitivity and specificity with real time analysis. However, the limitations are the regenerative ability or long –term usage of polymer/other materials. Single-analyte detection using contact-based sensing has remarkable applications, such as, real-time measurement of a molecule with high specificity. In this context, Förster resonance energy transfer (FRET)-based biosensors, bioluminescent resonance energy transfer, fluorescent-based, and surface plasmon resonance-based transducers have been introduced^97^ to improve specificity and sensitivity in terms of single molecule detection. Therefore, resonance energy transfer methods involving signal emission overlap detect multiple analytes. It is based on biomarkers between patients and their associated diseases. When micro-or-nano-cantilevers are used as transducers in electrochemical sensors, then biofabrication can also detect multiple analytes. However, most of the high sensitivity, real-time, and portable amperometric electrochemical biosensors have been developed for body fluid diagnosis.^98^ Optic-based biosensors represent the next major technology in biosensing, involving fiber-optic chemistry. Biological polymers can effectively detect the analyte using hydrogel-based crosslinking of monomers that are already hydrophilic in nature. Optical biosensors have been developed for DNA detection.^99,100^ Such biosensors give wider applications in biomedicine and forensic science. Additionally, the combination of biological materials such as enzymes/substrate, antibody/antigen and nucleic acids with additional incorporation of microorganisms, animal or plant cells and tissues, can contribute to the progression of optical biosensor technology. Recently, molecular optoelectronic systems have also shown promise in biometric recognition systems. In optical technology, both passive and active optical components can be placed on the same substrate for fabrication of multiple sensors on a single chip. Moreover, high quality polymer provides hybrid systems for optical biosensors.^4^

### 2.8 Challenges and future prospects in biosensor development

The development of biosensors brought forth a revolution in diagnostics, environmental monitoring, and biomedical research due to their outstanding sensitivity and specificity. Although there has been considerable improvements, several key challenges remain that limit the broader adoption and ability of biosensors to function to their full effect. These are the challenges faced, alongside some solutions, and they highlight a path forward for continuing to advance biosensor technology to address global demand.

A key challenge in developing biosensors is maintaining consistent sensitivity and specificity within complex biological matrices. Background noise and non-specific interactions between the matrix and the target analytes, as well as interferences from the matrix material, can drastically hinder the accurate detection of the specific analytes, particularly at low concentrations. This limitation can be overcome by designing more selective recognition elements such as engineered aptamers and peptide-based receptors, as well as new surface chemistries to limit non-specific binding. Techniques for signal amplification that use nanomaterials, such as plasmonic nanoparticles and 2D MXenes, are essential for increasing precision and detecting capabilities.

The cost-effectiveness and scalability of biosensor manufacturing pose a major obstacle. Metallic nanoparticles and graphene derivatives commonly improve performance, although they are often expensive and require complex manufacturing processes. Although laboratory prototypes serve as promising platforms for engineering new nanoscale pores, further research should focus on commercialization of such systems, which requires the early adoption of green chemistry methods for nanomaterial synthesis and modular and cost-effective designs enabling mass production without performance compromise. Breakthroughs in 3D printing and roll-to-roll manufacturing technologies hold promise for lowering production costs with high precision.

The integration of biosensors into point-of-care (POC) systems is a substantial difficulty. Point-of-care devices must be lightweight, intuitive, and adequately durable to function reliably in uncontrolled environments. Biosensors must be closely linked to microfluidic systems, portable energy sources, and analytical tools like artificial intelligence (AI) in order to accomplish these qualities. Smartphone apps with AI capabilities are excellent at improving diagnostic precision, automating data analysis, and giving consumers immediate feedback. Biosensors' compatibility with digital platforms determines their use in distributed and resource-constrained settings.

For biosensors applied in field or clinical settings, stability and reusability are particularly important. Environmental elements, including temperature fluctuations, humidity levels, and extended storage, could compromise the performance of biosensors. Operating lifetime of biosensors can be raised by means of strong materials with enhanced thermal and chemical stability, as well as protective coatings shielding fragile components. The development of reconfigurable or renewable sensing interfaces would help to promote reusability and thereby lowering running costs and waste.

Regulatory and validation barriers prevent biosensors from being quickly implemented in clinical and commercial settings. To meet regulatory requirements, biosensors—especially those used for diagnostics—must pass stringent testing. Standardising and harmonising validation methods across multinational jurisdictions is essential to accelerating market access without sacrificing efficacy or safety. This process can be accelerated, and the quick implementation of state-of-the-art biosensor technology in clinical settings made possible, by cooperation between academia, industry, and regulatory bodies.

Notwithstanding these obstacles, biosensor technology shows great potential because of breakthroughs in many areas and the inclusion of innovative fields. By allowing real-time data processing, predictive modelling, and adaptive calibration—which helps to alter biosensor performance—artificial intelligence and machine learning have great potential. MXenes and quantum dots are two advanced nanomaterials projected to raise biosensor sensitivity and extend their usage to many analytes. The development of wearable and implanted biosensors makes early disease identification and continuous health monitoring possible, thus transforming personalised treatment strategies. Moreover, the pursuit of ecologically sustainable biosensor production is in line with worldwide green technology objectives, thereby ensuring that diagnostic developments do not compromise ecological integrity.

## 3 Conclusion

Protein biosensors have changed diagnostics because they are very good at finding protein biomarkers that are important for diagnosing illnesses, keeping track of treatment, and advancing scientific research. Advanced nanomaterials, like carbon nanotubes, graphene derivatives, and MXenes, are much better at transmitting signals. This makes it possible to quickly and accurately find biomarkers in complex biological systems. Recent improvements in signal transduction methods have made biosensors more useful in more areas. For example, they can now be used to better track the immune system, treat infectious diseases, and find cancer, using methods such as electrochemical, optical, and CRISPR-based methods.

Even with these improvements, biosensor technology still has a few problems that need to be fixed before it can reach its full potential. Some of the biggest problems that need to be solved before widespread adoption is achieved are environmental sustainability, cost-effective production, legal compliance, and the ability to scale. It is essential to ensure that the designs of biosensors in point-of-care systems and remote diagnostic platforms are reliable, light, and compatible with AI-powered analytics in order for them to be successfully integrated. Addressing these challenges requires an interdisciplinary approach that integrates computer science, materials science, clinical medicine, and biotechnology.

Protein biosensors have a lot of potential because they are based on a lot of research and modern technology. Sustainable manufacturing methods, wearable diagnostics, real-time tracking systems, and multiplexing could change the way healthcare is provided around the world and how each person is treated. Protein biosensors may change the diagnostics industry by using new technologies to solve current limitations. They may provide substantial, accurate, and easily available solutions for healthcare and other fields.

## Data availability

No new data, software, or code were generated or analyzed in this study, as it is a review article. All data supporting the findings discussed in this manuscript are derived from previously published literature, which has been appropriately cited throughout. Any further details regarding specific datasets can be accessed through the original sources referenced in the manuscript.

## Author contributions

KJ: writing – original draft, resources, methodology, data curation. SS: software, writing – review and editing, visualization. DD: visualization, resources. MC: visualization, validation. SJ: visualization, resources. MS: visualization, validation. RH: writing – original draft, resources, formal analysis. RP: writing – original draft and review, resources, formal analysis, data curation, conceptualization. VC: writing – review & editing, writing – original draft, supervision, conceptualization.

## Conflicts of interest

There are no conflicts to declare.
